# Heterozygous FA2H mutations in autism spectrum disorders

**DOI:** 10.1186/1471-2350-14-124

**Published:** 2013-12-03

**Authors:** Isabelle Scheid, Anna Maruani, Guillaume Huguet, Claire S Leblond, Gudrun Nygren, Henrik Anckarsäter, Anita Beggiato, Maria Rastam, Fréderique Amsellem, I Carina Gillberg, Monique Elmaleh, Marion Leboyer, Christopher Gillberg, Catalina Betancur, Mary Coleman, Hiroko Hama, Edwin H Cook, Thomas Bourgeron, Richard Delorme

**Affiliations:** 1 Human Genetics and Cognitive Functions, Institut Pasteur, Paris, France; 2 CNRS URA2182, Paris, France; 3 APHP, Robert Debré Hospital, Child and Adolescent Psychiatry, Paris, France; 4 Gillberg Neuropsychiatry Centre, Gothenburg University, Göteborg, Sweden; 5 Department of Clinical Sciences, Lund University, Lund, Sweden; 6 Fondation FondaMental, French National Science Foundation, Creteil, France; 7 INSERM U955, Psychiatry Genetics, Créteil, France; 8 APHP, Robert Debré Hospital Paediatric Imaging, Paris, France; 9 Institute of Child Health, University College London, London, UK; 10 INSERM U952, Paris, France; 11 CNRS UMR7224, Paris, France; 12 UPMC Univ Paris 06, Paris, France; 13 Foundation for Autism Research, Sarasota, Florida 34235-7117, USA; 14 Department of Biochemistry and Molecular Biology, Medical University of South Carolina, Charleston, South Carolina, USA; 15 Institute for Juvenile Research, Department of Psychiatry, University of Illinois at Chicago, Illinois, USA; 16 University Denis Diderot Paris 7, 75013 Paris, France

**Keywords:** Autism, Brain, Gene, Myelin

## Abstract

**Background:**

Widespread abnormalities in white matter development are frequently reported in cases of autism spectrum disorders (ASD) and could be involved in the disconnectivity suggested in these disorders. Homozygous mutations in the gene coding for fatty-acid 2-hydroxylase (FA2H), an enzyme involved in myelin synthesis, are associated with complex leukodystrophies, but little is known about the functional impact of heterozygous *FA2H* mutations. We hypothesized that rare deleterious heterozygous mutations of *FA2H* might constitute risk factors for ASD.

**Methods:**

We searched deleterious mutations affecting *FA2H*, by genotyping 1256 independent patients with ASD genotyped using Genome Wide SNP arrays, and also by sequencing in independent set of 186 subjects with ASD and 353 controls. We then explored the impact of the identified mutations by measuring FA2H enzymatic activity and expression, in transfected COS7 cells.

**Results:**

One heterozygous deletion within 16q22.3-q23.1 including *FA2H* was observed in two siblings who share symptoms of autism and severe cognitive impairment, axial T2-FLAIR weighted MRI posterior periventricular white matter lesions. Also, two rare non-synonymous mutations (R113W and R113Q) were reported. Although predictive models suggested that R113W should be a deleterious, we did not find that FA2H activity was affected by expression of the R113W mutation in cultured COS cells.

**Conclusions:**

While our results do not support a major role for *FA2H* coding variants in ASD, a screening of other genes related to myelin synthesis would allow us to better understand the role of non-neuronal elements in ASD susceptibility.

## Background

Autism spectrum disorders (ASD) are characterized by impairments in reciprocal social interactions, language impairments and repetitive, stereotyped and ritualistic verbal and non-verbal behaviors. Beyond this unifying definition lies an extreme degree of clinical heterogeneity, ranging from mild to profound impairments [1,2]. Some of this heterogeneity might be due to heritable components of ASD arising from multiple independently regulated genes. One of these mechanisms is abnormal myelin development, which could explain the widespread abnormalities in white matter development that are thought to affect functional frontotemporal, frontolimbic, frontoparietal and inter-hemispheric connections in patients with ASD [3]. Indeed, transcriptomics coupled to pathway analyses have shown that immune-glial genes could contribute to the disorder [4]. Further evidence for connectivity abnormalities comes from dysregulation in certain genes, such as *CNTNAP2* and *CNTN4*, that code for proteins located at the nodes of Ranvier that organize the tight junctions between axons and myelinating glia [5]. It has been hypothesized that reduced long-range connectivity and excess local connectivity may affect system-wide integration of brain activity, which has been suggested to explain some symptoms of autism such as poor central coherence [6].

*FA2H* gene codes for fatty acid 2-hydroxylase (FA2H) [2], an enzyme that produces 2-hydroxylated fatty acids for incorporation into 2-hydroxydihydroceramide and 2-hydroxyceramide [7]. These ceramides, in turn, serve as precursors for the synthesis of galactosylceramide and sulfatides, essential lipid components of myelin sheaths. Homozygous mutations of the *FA2H* gene in humans are associated with three neurodegenerative disorders involving a wide variety of symptoms: complicated spastic paraplegia (SPG35) [8], leukodystrophy with spastic paraparesis and dystonia [9,10], and neurodegeneration associated with brain iron accumulation [11]. Although little is known about the effects of heterozygous mutations or deletions of *FA2H*, and its relations with ASD, we hypothesized that deleterious heterozygous mutations of *FA2H* might constitute a risk factor for this group of disorders. Using Genome Wide SNP arrays, we genotyped 1256 independent patients with ASD. We detected one heterozygous 167.1 kb deletion within 16q22.3-q23.1 including *FA2H*, in two siblings who share symptoms of autism and severe cognitive impairment, axial T2-FLAIR weighted MRI posterior periventricular white matter lesions. We then sequenced the *FA2H* gene in 186 patients with ASD and 353 controls, finding one non-synonymous mutation (R113W) shared by the affected siblings and transmitted by the mother who may carry a somatic mosaicism.

## Methods

### Sample

Nine hundred and ninety six patients from the Autism Genome Project (http://www.autismgenome.org) and 260 patients from the Paris Autism Research International Sibpair Study (PARIS) not included in the AGP, meeting stringent quality control (QC) criteria, were included in the study. Information concerning the phenotypic assessment of patients enrolled was described previously (for details see [12] and [13]). In brief, patients were recruited by the PARIS study at specialized clinical centers disposed in Paris (France) and Goteborg (Sweden). The Autism Diagnostic Interview-Revised (ADI-R) and Autism Diagnostic Observation Schedule (ADOS) were used for clinical evaluation and diagnosis. In Sweden, in some cases, the Diagnostic Interview for Social and Communication Disorders (DISCO-10) was applied instead of the ADI-R. Patients were included after a clinical and medical check-up with psychiatric and neuropsychological examination, standard karyotyping, fragile-X testing and brain imaging and EEG whenever possible. All patients were from Caucasian ancestry.

### Ethics statement

This study was approved by the local Institutional Review Board (IRB) and written inform consents were obtained from all participants of the study. The local IRB are the “Comité de Protection des Personnes” (Île-de-France Hôpital Pitié-Salpêtrière Paris) for France; the Sahlgrenska Academy Ethics committee, University of Gothenburg for Sweden. Written informed consent was obtained from all participating subjects. If the proband was under 18 years old, the proband's consent and written parental consent were obtained.

### CNV detection and validation

Two CNV calling algorithms, QuantiSNP and PennCNV, and the CNV viewer, SnipPeep (http://snippeep.sourceforge.net/) were used. To obtain high-confidence calls, the CNVs identified by QuantiSNP were validated by visual inspection of the Log R ratio and B allele frequency values. PennCNV was used to confirm inheritance status of the CNV calls.

CNVs were validated by quantitative PCR (qPCR) analysis using the Universal Probe Library (UPL) system from Roche. UPL probes and primers were designed using the UPL assay Design center from Rochar Applied Scicence. UPL probes were labeled with 6-FAM^TM^ fluorescein and the fluorescence was read with the Applied Biosystems 7500 Real-Time PCR System. Each assay was conducted in four replicates for target region probe-set and control region probe-set. Relative levels of region dosage were determined using the comparative CT method assuming that there were two copies of DNA in the control region. The relative copy number for each target region was calculated as 2^-ΔΔCT^ with confidence interval as 2^-(ΔΔCT±SD)^.

### DNA sequencing

The genomic structure of *FA2H* was obtained from http://genome.ucsc.edu/ (hg18). Primers spanning all seven intron-exon boundaries of *FA2H* were designed and used to amplify the regions of interest. Amplicons were produced from genomic DNA, and sequencing was performed using ABI 3730 DNA sequencer (Aplied Biosystems, Foster City, CA). Sequence comparison to reference sequence was performed using GenalysCarbon 2.8.2b (http://www.cng.fr).

### In silico protein function analysis

Multiple *in silico* applications were used to predict the functional effect of the amino acid substitutions, including SIFT (http://sift.jcvi.org/www/SIFT_seq_submit2.html), Polyphen-2 (http://genetics.bwh.harvard.edu/pph2/), and SNAP (http://rostlab.org/services/snap/).

### Cloning of PCR products

Exon 2 PCR products from genomic DNA derived from blood and buccal swabs were cloned using the StrataClone™ PCR cloning kit (Agilent Technologies). To determine the proportion of fragments in each PCR reaction containing either the mutant or the wild-type allele, DNA was isolated from single plasmid clones, analyzed by restriction enzyme using *Nco*I (New England Biolabs) and sequenced on an ABI 3100 genetics analyzer.

### FA2H enzyme activity assay and expression assays

Methods to measure FA2H enzyme activity were similar to those previously described [2]. Site-directed mutagenesis was performed using Phusion DNA polymerase (New England Biolabs, Ipswich, MA) and mutagenic primers per the manufacturer’s instructions and subcloned into pcDNA3 as previously described [9]. We sequenced the plasmid DNA twice to be absolutely sure that the mutation was present in the construct. COS7 cells were then transfected with pcDNA3, pcDNA3-hFA2H (WT), or pcDNA3-hFA2H (R113W). For the enzyme activity assay, cells were harvested 24 hours after transfection for lipid analysis. Various ceramide species were then quantified by liquid chromatography–mass spectrometry and normalized to protein content. For quantification of FA2H expression, the cells were harvested 24 hours after transfection and processed for western blot analysis using an anti-FA2H antibody (http://www.lsbio.com/).

## Results

### Molecular findings

#### A rare CNV is identified in the PARIS ASD patient cohort

**Figure 1:**
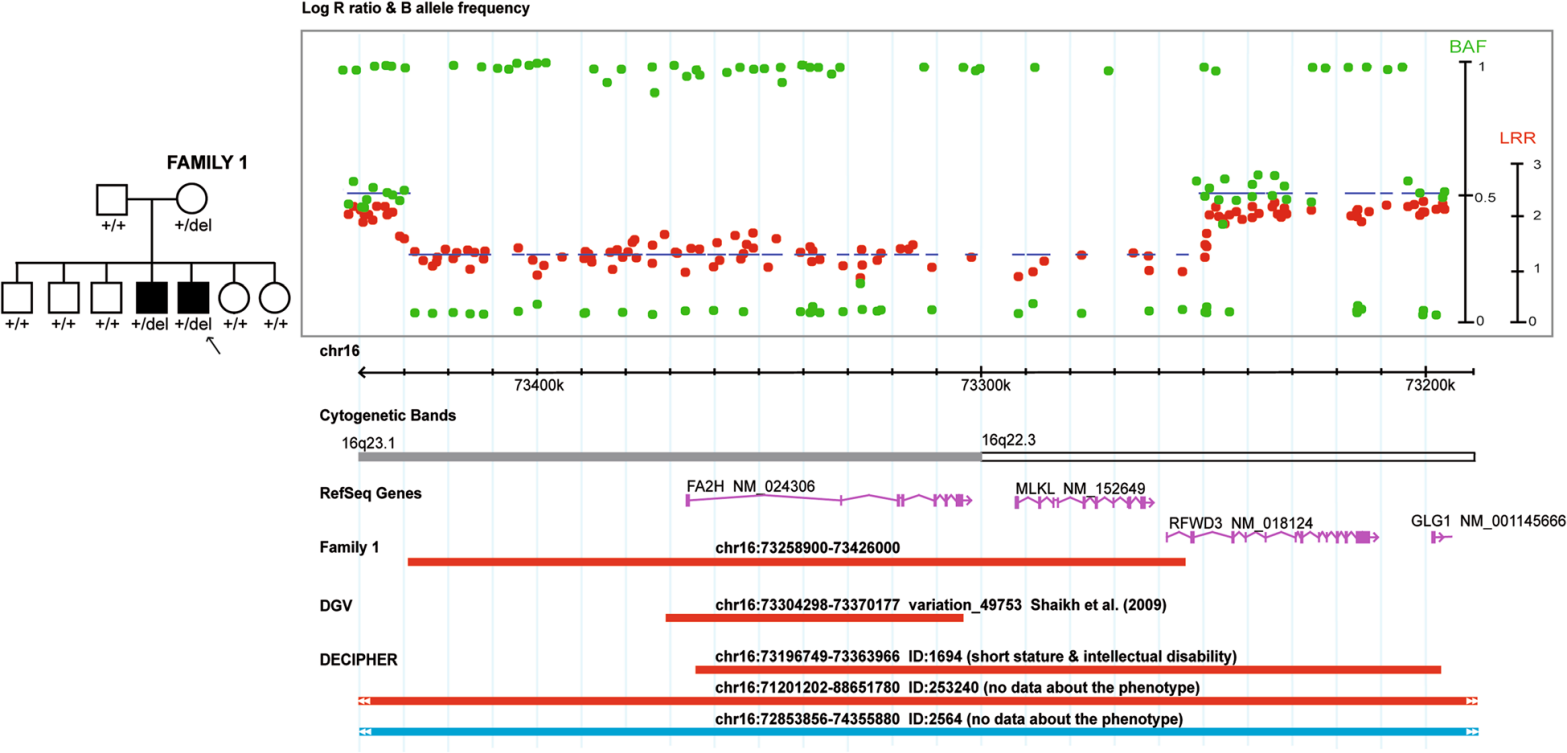
**An heterozygous *FA2H *deletion was identified with the Human 1 M-Duo SNP array from Illumina in the patient AUR139_7.** The heterozygous deletion, inherited from the mother and shared by an affected sibling, spans 167.1 kb on chromosome 16q22.3-q23.1, and included *FA2H*, *MLKL* and the first two exons of *RFWD3*. The upper plot shows Log R Ratio (in red) and B allele frequency (in green). QuantiSNP score is represented with a blue line and indicates the deletion size. One heterozygous deletion was previously referenced in the Database of Genomic Variants (chr16:73304298-73370177_hg18 variation_49753) and reported in 3/2026 children from the CHOP cohort. Three patients with developmental delay are reported in DECIPHER (https://decipher.sanger.ac.uk): two carried a deletion (ID: 1694, del chr16:73196749-73363966_hg18; ID: 253240, chr16:71201202-88651780_hg18) and one a duplication (ID: 2564, chr16:72853856-74355880_hg18). Red bars are deletions, and blue bars are duplications.

Among the 1256 independent patients with ASD genotyped using Illumina SNP arrays [996 from the AGP [12] and 260 from the PARIS study [13]], we detected a heterozygous 167.1 kb deletion within 16q22.3-q23.1 [chr16: 73258900-73426000_hg18] in one patient (AUR139_7) with autism and moderate intellectual disability (see clinical data section for details / Family 1). This CNV, inherited from the non-affected mother (Social Responsiveness Scale total score: 16: compared to a normal value <42; [14]) was shared by his affected sibling (AUR139_6) with autism and severe intellectual disability, and was absent from 5 unaffected siblings (Figure 1). The deletion, including *FA2H*, *MLKL* and the first two exons of *RFWD3*, was validated by qPCR analysis using DNA from an independent blood sample from both parents and the proband (data not shown). No additional rare micro-rearrangement was shared by the two affected siblings.

We then compared our results with those reported in databases. A similar heterozygous deletion was previously referenced in the Database of Genomic Variants (DGV; http://projects.tcag.ca/variation/) in 3/2026 healthy children recruited at the Children's Hospital of Philadelphia (CHOP) network (inheritance not mentioned). The DECIPHER database (https://decipher.sanger.ac.uk) contains only one patient (ID: 1694) with dysmorphism and intellectual disability/developmental delay who carried an inherited heterozygous deletion sharing at least 80% overlap with the deletion that we found (Figure 1). In this case, the deletion included *FA2H* (except the first exon); *MLKL* coding for a mixed lineage kinase domain-like protein; *RFWD3* coding for a ring finger and WD repeat domain protein; as well as the last exons of *GLG1*, which codes for a Golgi glycoprotein. Based on the Human Gene Expression Atlas 2 from the Genomics Institute of the Novartis Research Foundation (GNF; http://biogps.org/), only *FA2H* was reported to be expressed in the brain and be associated with cognitive dysfunctions.

#### An ASD-specific non-synonymous mutation in FA2H?

**Figure 2:**
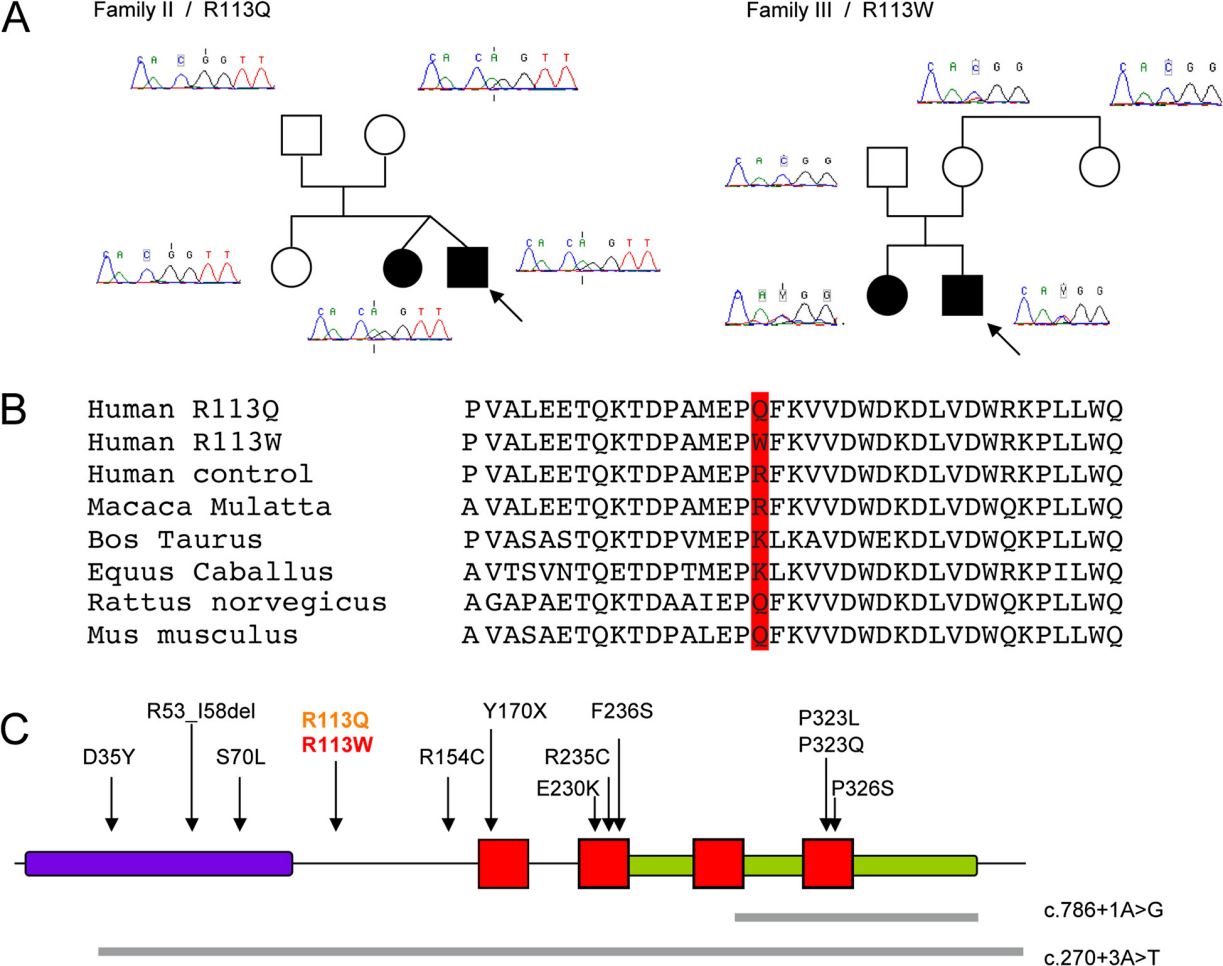
***FA2H *deleterious single nucleotide variants in our study and in literature. (A)** Distribution of R113Q in Family 2 and R113W in Family 3. In both families, mutations were shared by both affected siblings and inherited from a healthy parent. In Family 3, the sequence chromatograph of the mother showed a reduced signal for the mutation [the size of the allele T signal (in red) in the mother was twice lower than the signal observed in her affected children]. **(B)** We compared amino acid sequence conservation across species by aligning FA2H homologs using ClustalW (http://www.ebi.ac.uk/Tools/clustalw2/index.html). The R113 residue showed moderate conservation. **(C)** Localization of R113Q/R113W mutations and other reported mutations on a schematic representation of FA2H [8-11,15-18]. Red squares represent transmembrane domains, the purple rectangle represents the cytochrome B5-like heme-binding domain, and green rectangles represent the catalytic domain. The D35Y mutation has been reported in patients with leukodystrophy with spastic paraparesis and dystonia [9], the R235 and R53_I58del mutations in patients with hereditary spastic paraplegia [8], and the R154C and Y170X mutations in patients with degeneration associated with brain iron accumulation [11]. Splice site mutations predicted to result in skipping of exons 5-7(short gray line) and 2-7(long grey line) have been reported [9,15].

**Figure 3:**
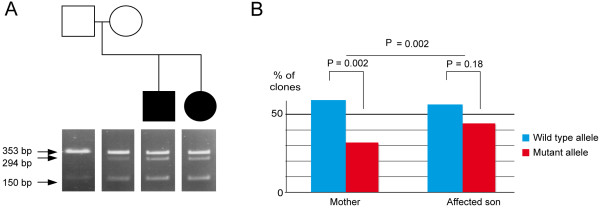
**Exploration of the somatic mosaicism in the Mother. (A)** Results of *Nco*I digestion of FA2H PCR products from DNA extracted from lymphocytes and buccal cells. A *Nco*I site is created by the R113W mutation in exon 2. In both types of tissue tested, we found a lack of digestion in the father, partial digestion in both affected children and a low level of partial digestion in the mother. **(B)** Percentage of clones carrying the wild type **(**allele C, blue bar) and mutant alleles (allele T, red bar) as determined by cloning PCR products amplified from blood DNA of the clinically affected patient and his unaffected mother (Family 3).

All *FA2H* exons were directly sequenced in a subset of patients with ASD (n = 186) from the Paris study and in controls (n = 353). Two non-synonymous variations, R113Q and R113W, affecting the same amino-acid in exon 2, were identified in Families 2 and Family 3 respectively. No additional deleterious mutation was found in 353 controls and on the remaining allele of *FA2H* in either affected sib-pair from Family 1. In Family 2, R113Q was shared by both affected sibs. This variant was transmitted by the healthy mother, and detected in one of our controls (Figure 2). *In silico*, the R113Q variant was predicted to be benign whatever the model of prediction used (Polyphen-2/prediction: benign, PSIC score difference: 0.002; SNAP/prediction: neutral, reliability index: 3, expected accuracy 78%; SIFT/prediction: tolerated, SIFT score median: 0.67 to 2.09). In contrast, R113W was predicted to be damaging (Polyphen-2/prediction: damaging, PSIC score difference: 0.992; SNAP/prediction: non neutral, reliability index: 2, expected accuracy 70%; SIFT/prediction: damaging, SIFT score median: 0.02 to 2.09). This mutation was not observed in our control samples and in the 1000 genomes database (http://browser.1000genomes.org/) but was reported in 0.08%(11/12985) of the individuals referenced in the Exome Variant Server (http://evs.gs.washington.edu/EVS/). The analysis of the segregation reported that R113W was shared by both affected siblings and inherited from the mother in Family 3. The sequence chromatograph of the mother showed a reduced signal for the mutation (similar results were obtained when sequencing the forward and reverse strands). To determine if this reduced signal could be related to a somatic mosaicism, exon 2 containing a *Nco*I site created by the mutation was amplified from DNA extracted from lymphocytes and buccal swabs. In both tissues tested, digestion of PCR products amplified from family members showed a lack of digestion in the father, a partial digestion in both affected children and also a low level of partial digestion in the mother (Figure 3), suggesting partial expression. To determine the proportion of fragments in each PCR reactions harboring either the mutant (T) or the wild-type (C) allele, blood DNA was isolated from single plasmid clones, analyzed by restriction enzyme using *Nco*I and sequenced. Among the 165 clones sequenced in the mother, 113 (68.5%) contained the C allele *vs.* 52 (31.5%) contained the T allele (binomial test, z = 2.96, *p* = 0.002), suggesting a degree of somatic mosaicism. In her affected son, used as control, we were unable to detect any significant skewed distribution of the C allele (n = 91, 55.8%) *vs*. the T allele (n = 72, 44.2%) (binomial test, z = 0.91 , *p* = 0.18). The allelic distribution in the mother differed significantly from that observed in her son (*X*^2^ = 5.6, *p* = 0.02). These data suggest that the mother in Family 3 exhibits somatic mosaicism for the R113W mutation, which could explain the lack of cosegregation between the variant and autistic symptoms in the pedigree. However, since we were unable to obtain a second sample of blood genomic DNA of the mother, and we did not directly measure the allele bias by quantitative PCR, we could not confirm these results.

#### The mutation does not impact FA2H enzymatic activity in transfected COS7 cells

We then assessed the impact of the R113W mutation on the enzymatic activity of FA2H in COS7 cells *in vitro*. Liquid chromatography/mass spectrometry analysis of COS7 cells transfected with pcDNA3-hFA2H R113W demonstrated no significant difference in enzyme activity from WT cells (Additional file 1: Figure S1). Major 2-hydroxy ceramide species, a product of FA2H activity, were significantly elevated by expression of either wild type or R113W FA2H, indicating that the mutation did not affect catalytic activity of FA2H. In addition, western blot analysis of COS7 cells transfected with the same mutant or wild-type FA2H constructs demonstrated no significant difference in overall FA2H expression (Additional file 1: Figure S1).

### Clinical information

#### Family 1

A 5-year-old boy *(AUR139_6)* was referred with his 4-year-old brother *(AUR139_7)* to the child psychiatry department (Robert Debré Hospital, Paris) due to severe developmental delay. Both of them were born at term (39 and 40 weeks) to non-consanguineous Caucasian parents after uneventful pregnancies and deliveries. Both parents reported psychiatric disorders. The father had symptoms of anxiety in the context of schizotypal personality traits. One of the father’s brothers (among 10 siblings) might have an intellectual disability, but a direct assessment was not possible. The mother described recurrent episodes of major depressive disorders during the previous ten-year period. Clinical psychiatric examination yielded normal results. She had left her biological family during childhood, and was unable to provide additional information about her extended family. Of the five additional siblings of the index cases, one had social phobia that emerged during childhood, and two of the others had attention-deficit/hyperactivity disorder (ADHD).

**Figure 4:**
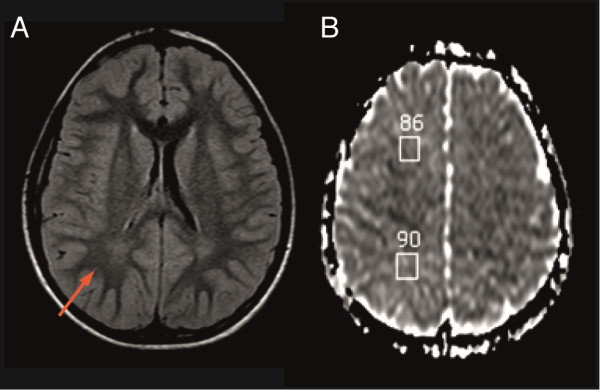
**Brain MRI of the patient AUR139_7 at 9 years of age. (A)** Axial T2-FLAIR weighted images showed slightly prolonged relaxation times in the periventricular white matter, more prominent in the posterior region (red arrow) with no significant loss of volume. **(B)** Axial diffusion-weighted images (DWI) showed slightly elevated apparent diffusion coefficient (ADC) values.

Birth weight and length of both index patients were in the normal range, but occipitofrontal head circumference (OFC) of both siblings was > 1 standard deviation (SD) below the mean. During early development, pediatricians reported moderate hypotonia and difficulty in establishing eye contact. Speech was severely delayed, and neither of them had acquired functional language at time of evaluation. Both boys were diagnosed with autism according to the Autism Diagnostic Interview-Revised (ADI-R) and the Autism Diagnostic Observation Scale (ADOS). The youngest brother was more severely “autistic” according to his performance on the Childhood Autism Rating Scale (CARS) (43 vs. 35). Cognitive performance of both brothers as measured by the Raven’s Progressive Matrices was below the first percentile. When examined at the time of inclusion in the study, growth parameters were within the normal range for both patients with the exception of the OFC, which remained > 1 SD below the mean. The neurological exams of both patients were normal except for moderate localized ligament laxity. The oldest brother exhibited minor signs of dysmorphism, with brachymetatarsia of the 4th and 5th fingers, arachnoid fingers and widely spaced nipples. The younger brother also displayed minor signs of dysmorphism including a triangular face, a high forehead, long fingers, and bilateral covovarus feet. The brain MRI of the younger patient AUR139_7 at 9 years of age showed a mild T2-FLAIR hypersignal located in the posterior periventricular region (Figure 4) with no significant loss of volume. Axial diffusion-weighted images (DWI) produced slightly elevated apparent diffusion coefficient (ADC) values: 0.86 in the frontal region and 0.9 in the parieto-occipital region (compared to a normal value of 0.8). A brain MRI performed on the older brother showed a similar hyperintensity signal on the axial T2-FLAIR weighted images, located in the posterior periventricular region. High-resolution karyotype, fragile X testing and metabolic screening (urine amino acids, mucopolysaccharides, organic acids) were normal in both brothers.

#### Family 2

The patient was a 15-year-old male, born at term after an uneventful twin pregnancy and delivery (38th week) to non-consanguineous Caucasian parents. The maternal personal and family history revealed no prior instances of mental or neurological disorders, except for a possible post-partum depression after the twin pregnancy for which she did not receive any treatment. The clinical psychiatric examination produced normal results. The father reported a general anxiety disorder that emerged during childhood in the context of a diagnosis of ADHD. At the time of the assessment, only the anxiety disorder persisted. His clinical exam was normal. The older sister, aged 17 years at the time of the assessment, did not report any psychiatric disorder but had experienced an idiopathic pubertal delay. Her clinical exam was normal except for mild strabismus. Birth weight of the patient was 3170 g (25th percentile), length 50 cm (49th percentile), and OFC 36 cm (53rd percentile). Apgar scores were 10 and 10 at 1 and 5 min after birth. Despite normal development during infancy, including walking at 15 months, the parents reported a pronounced intolerance to frustrations and noises at age 2. They also noted a paucity of interactions accompanied by stereotyped body movements. The patient exhibited severely delayed speech, with first words at 4 years of age. Referred to a psychiatric unit at the age of 5 years, he was diagnosed with autism, based on DSM-IV and ICD-10 criteria. He also met criteria for autism according to the ADI-R and the Autism Diagnostic Observation Scale (ADOS) [19,20]. Assessment of his cognitive performance with the Raven’s Colored Progressive Matrices test indicated that his intelligence quotient (IQ) was below the first percentile for his age. When examined at age 15 years, his height, weight and OFC were all in the normal range, but he had minor dysmorphic features including a long and narrow face, arachnoid fingers and a supernumerary nipple. The neurological exam was normal. His expressive language was limited to restricted and stereotyped sentences. His dizygotic twin sister who shared the *FA2H* mutation also exhibited a severe intellectual disability with autistic features, but also displayed a severe psychomotor delay and microcephaly. The clinical presentation was very different from that of her twin brother, and suggested a Rett syndrome. Screening of the *MECP2* gene at the age of 4 years, showed a *de novo* deletion (c.1126del50) in exon 3 resulting in the truncation of the protein. None of the other members of the family carried this deletion.

#### Family 3

A 5-year old male patient, the second child of non-consanguineous Caucasian parents with no family history of neurodevelopmental disorders, was born at term after uneventful pregnancy and delivery. While little information is available about his early development, at the time of the inclusion he was diagnosed with autism based on DSM-IV and ICD-10 criteria. He also met criteria for autism according to the ADI-R. He displayed severe intellectual disability although standard IQ assessment with the Stanford-Binet test was difficult. The clinical examination of the patient produced normal results except for one small café-au-lait spot on his skin. At age 19, the patient developed generalized seizures, which were subsequently easily controlled on topiramate. The clinical presentation of his older sister, aged 6 years was very similar. She also met criteria for autism and moderate intellectual disability (Stanford-Binet test, full scale IQ: 43). Her clinical physical examination was also within the normal range with the exception of one small café-au-lait spot on her skin. Like her brother, she developed generalized seizures at age 22 that were efficiently controlled on topiramate. The healthy mother also had one small café-au-lait spot on her skin.

## Discussion

Recent work has shown that homozygous mutations in *FA2H* can produce a variety of severely impaired neurological phenotypes. In this study, we investigated the role of heterozygous *FA2H* mutations and deletions in the development of ASD. We described two affected siblings from our PARIS cohort with severe autism and intellectual disability who present axial T2-Flair weighted MRI posterior periventricular white matter hypersignals accompanied by a rare heterozygous deletion of 16q22.3-q23.1 overlapping *FA2H*. No additional patients with a CNV encompassing *FA2H* were identified among the 996 AGP patients with ASD [12] or the additional sample from the PARIS cohort (n = 260) (1/1256, 0.08%). In the CHOP cohort, three out of 2026 control children exhibited a heterozygous deletion of *FA2H* (0.15%). However, none of those children have been clinically evaluated for the absence of autistic symptoms [21]. Nonetheless, one subject in the DECIPHER database who carried a similar heterozygous deletion encompassing *FA2H* had also intellectual disability and hypotonia.

Although several models predicted that R113W should be a deleterious mutation, we did not find that this mutation affected FA2H enzymatic activity as measured in an over expression model *in vitro*. This assay, however, might have been unable to detect subtle reductions in enzymatic activity. Among the non-synonymous deleterious mutations previously reported in *FA2H-*related disorders, only D35Y had a striking effect on FA2H enzymatic activity [9]. R154C did not affect significantly the enzyme activity when tested in a similar *in vitro* model (although the mutation was involved in neurodegeneration with brain iron accumulation), and R235C had a moderate effect when over-expressed [8]. Notably, R113W and R154C are both located in functionally uncharacterized regions of the FA2H protein (Figure 2) that could explain their lack of *in vitro* impact (in contrast to D35Y and R235C located in functional domains that have a clear *in vitro* impact on *FA2H* activity).

Based on our results, heterozygous *FA2H* mutations do not seem to play a major role in the etiology of ASD. This may be consistent with previous publications describing patients with homozygous mutations in *FA2H*, which do not report an increased prevalence of autistic or psychiatric symptoms in the relatives carrying the heterozygous mutations. However, the role of *FA2H* heterozygous mutations as minor risk factors for ASD would need additional considerations. Specifically, exome sequencing of the probands and their parents would have helped to determine if additional mutations in genes related to autism or intellectual disability or in the same pathway than *FA2H*, might be involved in the families we presented. Recent findings suggested that co-occurrence of deleterious mutations, together with inherited variations plays a major role in the genetic susceptibility to ASD [13,22].

Finally, the case studies described here may support the growing consensus that proteins involved in myelination, and not only those involved in neuronal function, might be important in the etiology of ASD [23]. A recent post mortem study of white matter in the brains of ASD patients revealed a decreased number of highly myelinated long-distance axonal projections in the anterior cingulate cortex accompanied by over-expression of Growth Associated Protein 43 and abnormally high numbers of thin axons involved in local connectivity [24]. This study also reported decreased myelin thickness in axons located in the orbitofrontal cortex. According to the hypothesis of frontal dis-connectivity in ASD [6], altered white matter composition in anterior cingulate cortex and orbitofrontal cortex could change the functional relationship between prefrontal areas as well as transmission to downstream regions. Results from diffusion tensor imaging tractography used to measure white matter in ASD patients also support this hypothesis [25,26]. Specifically, ASD patients had a smaller total white matter volume, lower fractional anisotropy of white matter tracts connecting the putamen to frontal cortical areas, and higher mean diffusivity of white matter tracts connecting the nucleus accumbens to frontal cortex [26].

## Conclusion

In summary, the evidence implicating white matter abnormalities in ASD suggest that additional genes or environmental factors involved in myelination take part in the development of these disorders. A screening of other genes related to myelin synthesis would allow us to better understand the role of these non-neuronal elements in ASD susceptibility.

## Abbreviations

ADC: Apparent diffusion coefficient; ADHD: Attention deficit-hyperactivity disorder; ADI-R: Autism diagnostic interview-revised; ADOS: Autism diagnostic observation scale; ASD: Autism spectrum disorder; CARS: Childhood autism rating scale; CHOP: Children’s Hospital of Philadelphia; CNV: Copy number variant; COS: CV-1 (simian) in Origin, and carrying the SV40 genetic material; DGV: Database of genomic variants; DNA: Deoxyribonucleic Acid; DWI: Diffusion-weighted images; FA2H: Fatty-acid 2-hydroxylase; MRI: Magnetic resonance imaging; OFC: Occipitofrontal head circumference; PCR: Polymerase Chain Reaction; PSIC: Position-specific independent counts; QC: Quality control; SD: Standard deviation; SIFT: Sorting tolerant from intolerant; SNAP: Screening for non-acceptable polymorphisms; SNP: Single nucleotide polimorphism; SPG35: Spastic paraplegia autosomal recessive 35; UPL: Universal probe library; WT: Wild type.

## Competing interests

The authors declare they have no competing interests.

## Authors’ contribution

IS, MA, GH, CL, GN, HA, PC, MR, FA, ICA, ME and AB helped to collect the data. CG, CB, TB, MC, HH, EDC and RD interpreted the results, drafted the manuscript. CB, CG, ML, MC, EHC, TB and RD were the main investigators. IS, MA and HH performed the molecular experiments. All authors read and approved the final manuscript.

## Pre-publication history

The pre-publication history for this paper can be accessed here:

http://www.biomedcentral.com/1471-2350/14/124/prepub

## Supplementary Material

Additional file 1: Figure S1 (A) FA2H expression in transfected COS7 cells. COS7 cells were transfected with pcDNA3, pcDNA3-hFA2H (wild type WT), or pcDNA3-hFA2H (R113W). Cells were harvested 24 hours after transfection and processed for Western blot. The expression of FA2H did not appear significantly different between WT and R113W mutant. (B) Ceramide levels in transfected COS7 cells. COS7 cells were transfected with pcDNA3, pcDNA3-hFA2H (wild type: WT), or pcDNA3-hFA2H (R113W). Cells were harvested 24 hours after transfection for lipid analysis. Various ceramide species [with 2-hydroxy fatty acid *(B1)* or with unsubstituted fatty acid *(B2)*] were quantified by liquid chromatography–mass spectrometry and normalized to protein contents. Major 2 hydroxy-ceramide species were significantly elevated by expression of either WT (black bars) or R113W FA2H (red bars) when compared to pcDNA3 only (white bars), whatever the length of the fatty acid chain, indicating that R113W mutation did not affect the catalytic activity of FA2H. Click here for file
