# Supplementary figures and images for: Heterozygous FA2H mutations in autism spectrum disorders
Source: BMC Med Genet. 2013 Dec 3;14:124. doi: 10.1186/1471-2350-14-124 (PMC4219428; doi:10.1186/1471-2350-14-124)

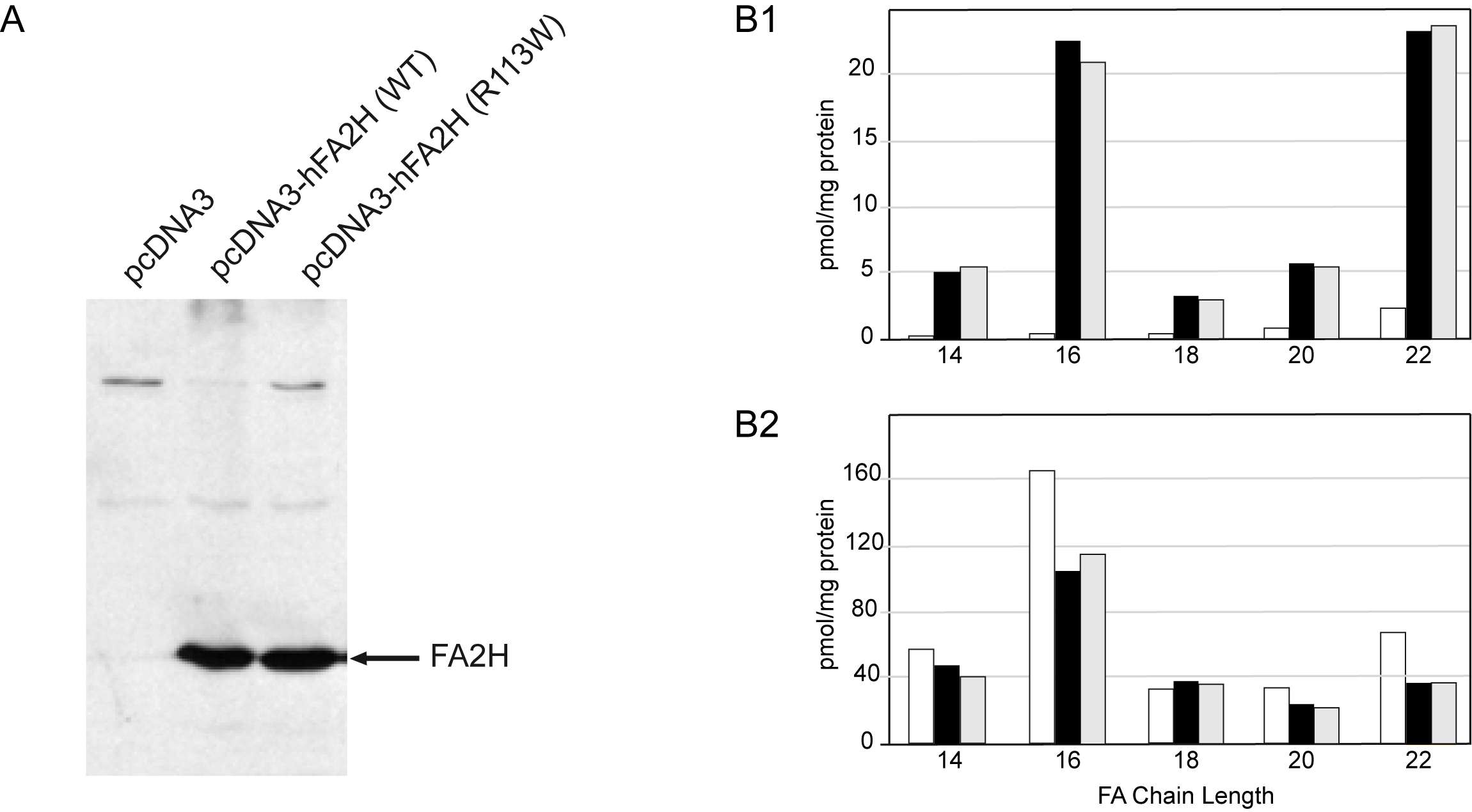

Supplement: Additional file 1: Figure S1 — (A) FA2H expression in transfected COS7 cells. COS7 cells were transfected with pcDNA3, pcDNA3-hFA2H (wild type WT), or pcDNA3-hFA2H (R113W). Cells were harvested 24 hours after transfection and processed for Western blot. The expression of FA2H did not appear significantly different between WT and R113W mutant. (B) Ceramide levels in transfected COS7 cells. COS7 cells were transfected with pcDNA3, pcDNA3-hFA2H (wild type: WT), or pcDNA3-hFA2H (R113W). Cells were harvested 24 hours after transfection for lipid analysis. Various ceramide species [with 2-hydroxy fatty acid (B1) or with unsubstituted fatty acid (B2)] were quantified by liquid chromatography–mass spectrometry and normalized to protein contents. Major 2 hydroxy-ceramide species were significantly elevated by expression of either WT (black bars) or R113W FA2H (red bars) when compared to pcDNA3 only (white bars), whatever the length of the fatty acid chain, indicating that R113W mutation did not affect the catalytic activity of FA2H. [file 1471-2350-14-124-S1.tiff]
